# β-Aminobutyric acid promotes stress tolerance, physiological adjustments, as well as broad epigenetic changes at DNA and RNA nucleobases in field elms (*Ulmus minor*)

**DOI:** 10.1186/s12870-024-05425-6

**Published:** 2024-08-15

**Authors:** Hans Hoenicka, Susanne Bein, Marta Starczak, Wolfgang Graf, Dieter Hanelt, Daniel Gackowski

**Affiliations:** 1 Thünen Institute of Forest Genetics, Sieker Landstr. 2, D-22927 Grosshansdorf, Germany; 2 Institute of Plant Science and Microbiology, University of Hamburg, Ohnhorst. 18, D-22609 Hamburg, Germany; 3 Department of Clinical Biochemistry, Faculty of Pharmacy, Collegium Medicum in Bydgoszcz, Nicolaus Copernicus University in Toruń, Karlowicza 24, Bydgoszcz, 85-095 Poland

**Keywords:** β-Aminobutyric acid, Drought stress, Epigenetics, Forest trees, Holoepigenome, Hologenome, Non-canonical nucleobases, Plant memory, Priming, Stress tolerance

## Abstract

**Background:**

β-Aminobutyric acid (BABA) has been successfully used to prime stress resistance in numerous plant species; however, its effectiveness in forest trees has been poorly explored thus far. This study aimed to investigate the influence of BABA on morphological, physiological, and epigenetic parameters in field elms under various growth conditions. Epigenetic changes were assessed in both DNA and RNA through the use of reversed-phase ultra-performance liquid chromatography (UPLC) coupled with sensitive mass spectrometry.

**Results:**

The presented results confirm the influence of BABA on the development, physiology, and stress tolerance in field elms. However, the most important findings are related to the broad epigenetic changes promoted by this amino acid, which involve both DNA and RNA. Our findings confirm, for the first time, that BABA influences not only well-known epigenetic markers in plants, such as 5-methylcytosine, but also several other non-canonical nucleobases, such as 5-hydroxymethyluracil, 5-formylcytosine, 5-hydroxymethylcytosine, N6-methyladenine, uracil (in DNA) and thymine (in RNA). The significant effect on the levels of N6-methyladenine, the main bacterial epigenetic marker, is particularly noteworthy. In this case, the question arises as to whether this effect is due to epigenetic changes in the microbiome, the plant genome, or both.

**Conclusions:**

The plant phenotype is the result of complex interactions between the plant’s DNA, the microbiome, and the environment. We propose that different types of epigenetic changes in the plant and microbiome may play important roles in the largely unknown memory process that enables plants to adapt faster to changing environmental conditions.

**Supplementary Information:**

The online version contains supplementary material available at 10.1186/s12870-024-05425-6.

## Background

Current global warming, longer drought periods, and a worldwide spread of insects and diseases all represent serious threats to the integrity of forests worldwide [1]. As a consequence, many tree species are abruptly facing new abiotic and biotic stress factors for the first time. Although resistance breeding can help overcome these stress factors, the resistant individuals required for breeding are often missing [2]. Furthermore, the prolonged non-reproductive phase of trees, lasting between five and forty years [3], additionally hampers resistance induction with conventional breeding methods when compared to annual plants [4]. Genetic modification and genome editing have a potential for fast resistance induction in trees [5], however both methods are still banned for applied research in most countries [6]. Therefore, the development of alternative methods for faster induction of stress resistance in tree species is of paramount importance.

Priming approaches have a potential for the induction of stress tolerance in trees. Priming is characterized by an improved activation of defense mechanisms. Several methods have been successfully used to promote priming in different plant species (rev. in: [7]). Biotic stimuli from pathogens, endophytes, or plagues, as well as abiotic factors such as chemicals and temperature, can increase resistance in plants by acting as warning signals. Biotic priming is mostly involved in improving biotic stress tolerance, whereas abiotic priming is usually related to improved abiotic stress tolerance. However, there are exceptions to this rule, as some priming agents like β-aminobutyric acid (BABA) can promote both abiotic as well as biotic stress tolerance [7]. Priming stimulus perception may lead to many changes in the plant at metabolic, transcriptional, epigenetic, and physiological levels that can promote a primed state characterized by an increased resistance. Different abiotic and biotic methods have been used for resistance priming in plants (rev. in: [7–10]). The non-proteinogenic amino acid BABA, one of the most used priming molecules (rev. in [11]), has two isomers: α-aminobutyric acid (AABA) and γ-aminobutyric acid (GABA). Both GABA and BABA can be naturally induced by stress in plants [12, 13]. GABA, but not AABA, has been also successfully used for priming resistance in plants [13–15]. BABA allowed resistance induction against a high number of biotic and abiotic stress factors in more than 40 different plant species [11]. Compared to annual plants there are few reports on priming in forest trees [16–28], with two of them involving field elms [25, 26], and only two reports with BABA as the priming agent [27, 28].

The maintenance of improved stress tolerance is key for surviving rapidly changing environmental conditions. Priming-induced stress resistance can be a short-term response but also long-term priming [29–33], maintained throughout the plant’s life cycle, has been reported. This is especially important for long-living plant species like forest trees that can live for hundreds of years. In this sense, there are some reports of long-term priming in trees [16, 34]. Furthermore, a transmission of the priming state to subsequent generations has been confirmed in annual [35–42], and tree species [22].

Plants lack cognitive abilities based on brains and other neural structures. However, they show complex behaviors, e.g. stress memory and decision making, which allow them to cope with the challenge of continuous exposure to changing environmental conditions (rev. in: [43, 44]). Plant memory seems to be regulated by epigenetic changes and other factors which modulate gene activity and coordinate responses to stress conditions [45–50]. Furthermore, those epigenetic changes can sometimes be inherited to the next generations [51–54].

Modified amino acids and nucleobases are the basis of epigenetic regulation in protein (epiproteomic), DNA (epigenomic), and RNA (epitranscriptomic) in animals and plants (rev. in: [55, 56]). Epigenetic modifications promote functional diversity that allows canonical nucleobases to gain new functions in both DNA and RNA [57]. Those modifications can influence DNA and RNA structure, and change their interactions with other molecules, specially proteins [58]. More than 52 DNA ([59]; https://dnamod.hoffmanlab.org/) and 163 RNA ([57], http://modomics.genesilico.pl) nucleobase variants have been reported in different organisms, but only some of them have been hitherto recognized as epigenetic markers in plants [60]. The best studied modified deoxynucleoside and epigenetic marker in plants is 5-methyl-2’-deoxycytidine (5-mdC). In addition to methylation of cytosine, other epigenetic changes such as oxidation of 5-mdC to 5-(hydroxymethyl)-2’-deoxycytidine (5-hmdC), 5-formyl-2’-deoxycytidine (5-fdC), 5-carboxy-2’-deoxycytidine (5-cadC), and methylation of 2’-deoxyadenosine to N6-methyl-2’-deoxyadenosine (N6-mdA), have been identified in plants [55].

Modified ribonucleosides have been reported in tRNAs, mRNA, rRNA, small nucleolar RNA (snoRNA) and small nuclear RNA (snRNA), with tRNAs exhibiting the highest number of known modifications [61, 62]. N6-methyladenosine (N6-mrA) is the most common nucleoside modification of mRNA in eukaryotic cells. It has been correlated to regulation of embryo development, flowering-time control, and stress response in plants [63]. A drastic reduction in the ribonucleoside variation was reported in etiolated wheat leaves [64].

Most epigenetic studies on forest trees species have focused on 5-mdC in DNA (rev. in [65]). However, the presence of 5-(hydroxymethyl)-2′-deoxyuridine (5-hmdU), 5-hmdC and 5-fdC was also confirmed in Norway spruce buds [66]. First epitranscriptomic studies have revealed the presence of N6-mrA in trees [67, 68].

In this study, the effect of BABA was studied in field elms, which is a strongly endangered tree species [69]. Our results confirmed that BABA promotes drought tolerance and induces various modifications in the growth and physiology of field elms. In a subsequent part of this study, various effects of these treatments on the epigenome and epitranscriptome were confirmed. The detected correlation between the levels of BABA and non-canonical nucleobases suggests that both might be key players of a stress memory system building the basis for faster adaptation in plants. These findings are valuable for developing new strategies of resistance induction adapted to forest trees and other plant species.

## Results

### BABA increased drought stress tolerance

**Fig. 1:**
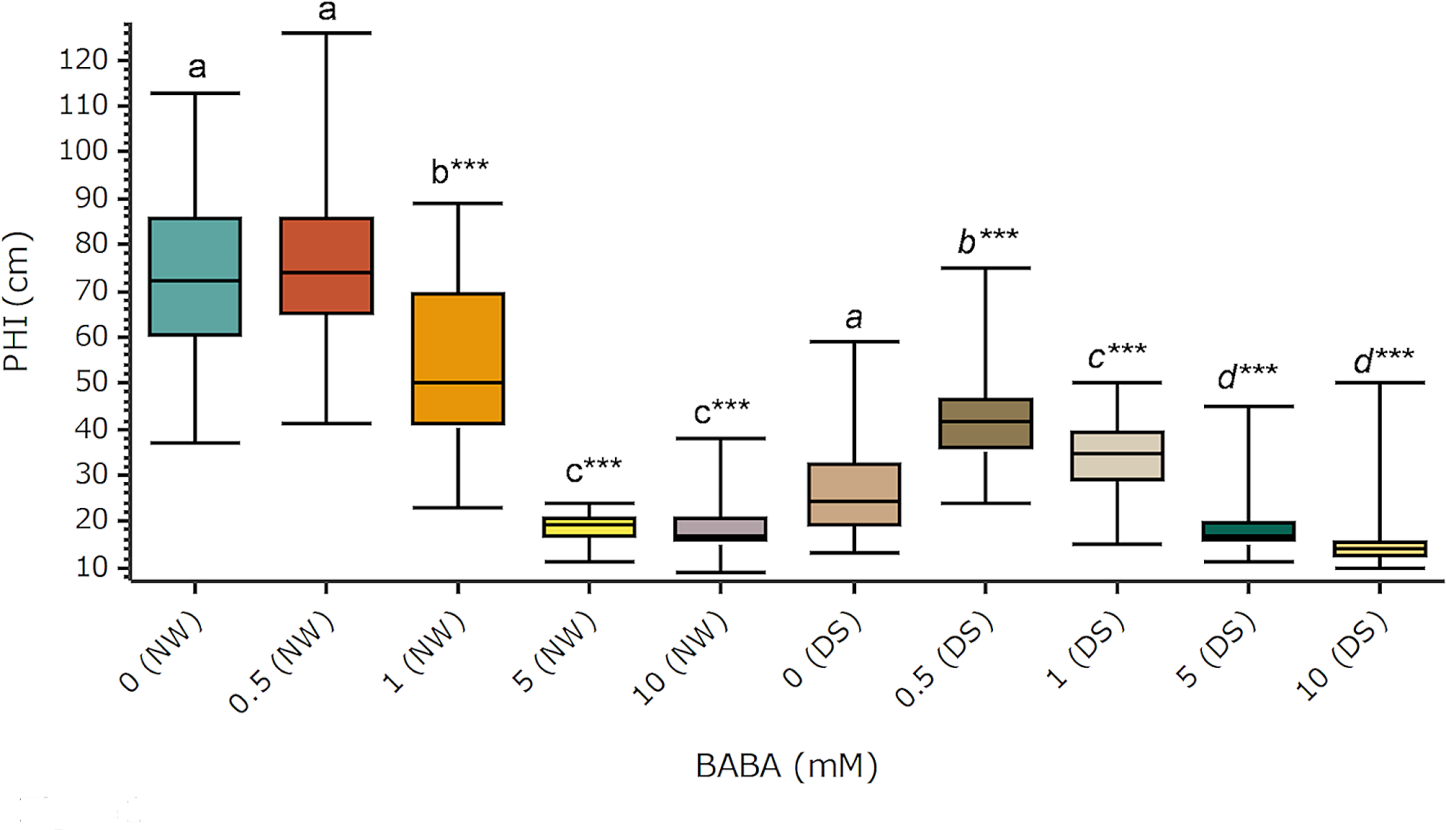
Influence of BABA on plant growth. Treatments were carried out by drenching and spraying four-month-old elm greenhouse seedlings with different BABA concentrations. Plants were kept under normal watering (NW) or drought stress (DS) conditions during 8 weeks. Plant height increase (PHI) during stress tests was measured. Box plots present median, upper and lower quartiles; whiskers show minimum and maximum values, *n* = 30. Statistical data analyses: Kruskal Wallis test and multiple comparisons of Nemenyi. Comparisons were made between treatment groups and the corresponding watering control group, 0 (NW) or 0 (DS). Statistically significant differences (*p* < 0.05) are shown with different letters above groups. *P*-values respect to controls are shown: *p < 0.05, **p < 0.01, ***p < 0.001

The plant height increase (PHI) after 8 weeks under drought stress conditions (DS) was 60% (0.5 mM BABA, *p* < 0.001) and 29% (1 mM BABA, *p* < 0.05) compared to the DS control group (0 mM BABA, DS) (Fig. 1). Increased PHI under DS suggests that BABA promoted DS tolerance in elms. A similar PHI was not obtained under normal watering conditions (NW) (Fig. 1). The plant height (PH) at the end of experiments was not significantly influenced by 0.5 mM BABA under NW, and 0.5- and 1-mM BABA under DS conditions (Supporting Information Fig. S4 and S5). However, higher BABA concentrations significantly decreased PHI and PH under both watering conditions (Fig. 1, Supporting Information Fig. S4 and S5).

### BABA treatment led to changes in concentration of leaf pigments and photosynthetic activity

**Fig. 2:**
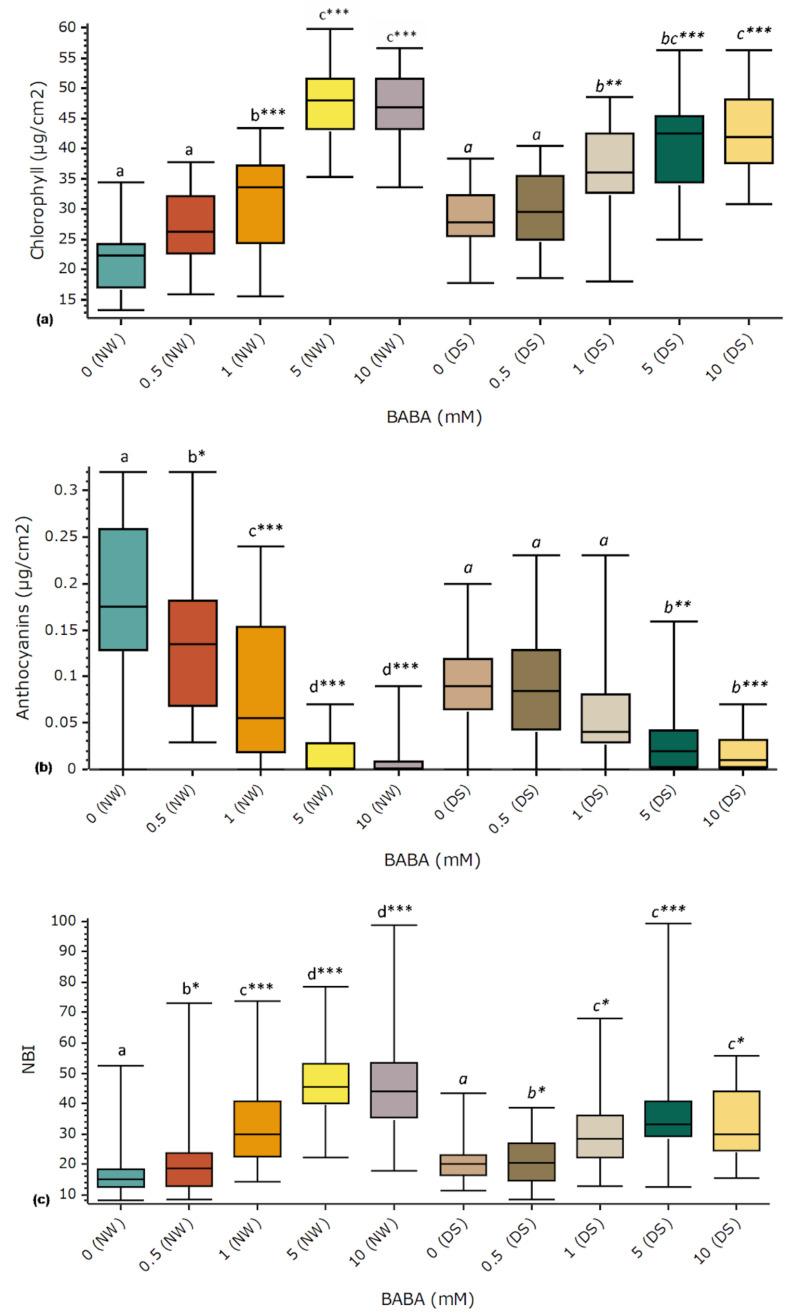
Influence of BABA on leaf pigments concentrations and NBI index. Treatments were carried out by drenching and spraying four-month-old elm greenhouse seedlings with different BABA concentrations. Plants were kept under normal watering (NW) or drought stress (DS) conditions during 8 weeks. Results are based on measurements of the epidermal UV absorbance of chlorophyll (**a**), anthocyanins (**b**) and NBI (**c**) with a Dualex device. Uppermost leaves were measured at the end of the experiments. Box plots present median, upper and lower quartiles; whiskers show minimum and maximum values, *n* = 30. Statistical tests: Statistical data analyses: Kruskal Wallis test and multiple comparisons of Nemenyi. Comparisons were made between treatments groups and the corresponding watering control group, 0 (NW) or 0 (DS). Statistically significant differences (*p* < 0.05) are shown with different letters above groups. *P*-values respect to controls are shown: *p < 0.05, **p < 0.01, ***p < 0.001

**Fig. 3:**
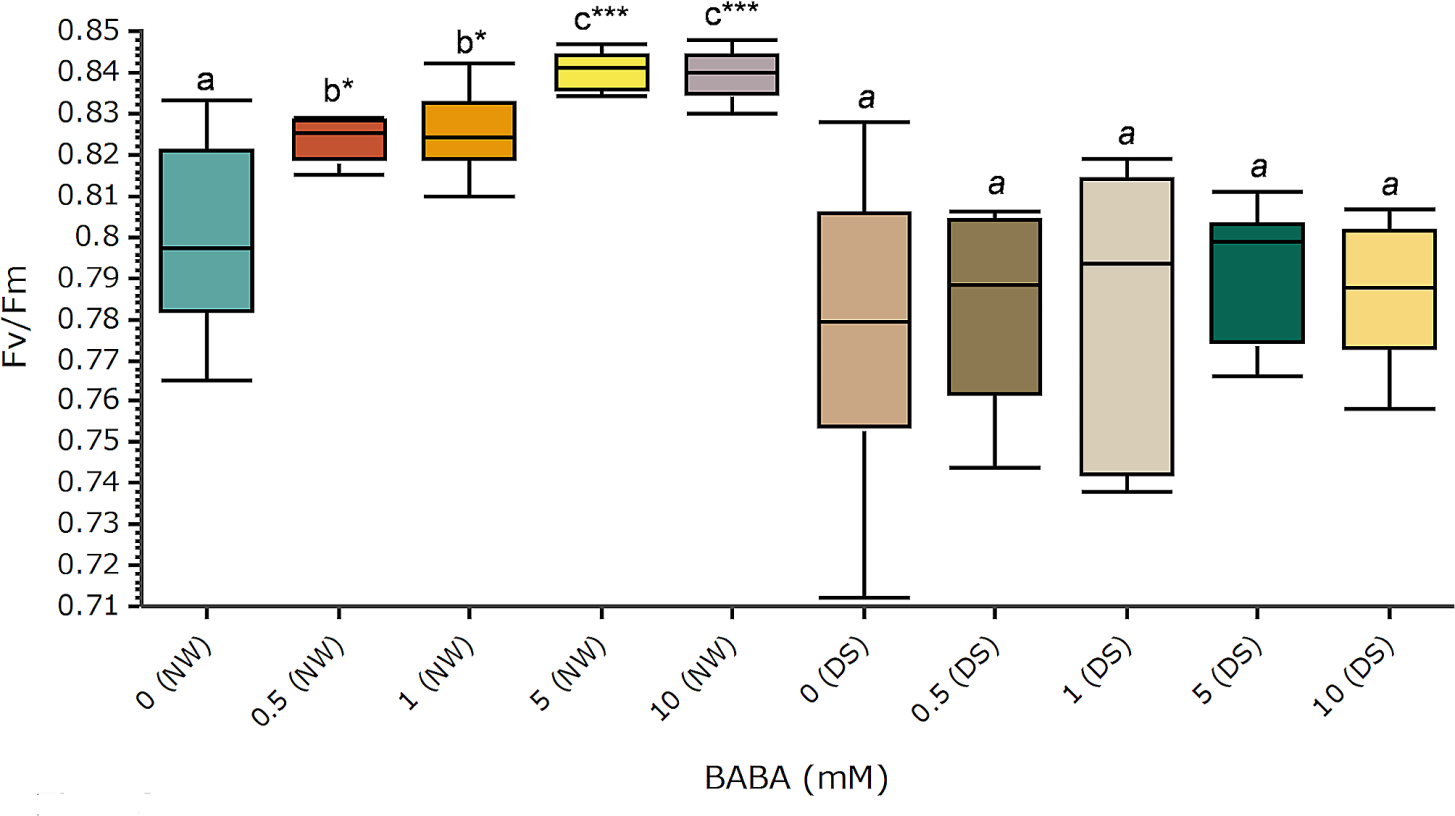
Influence of BABA on the maximum quantum yield of PSII photochemistry (Fv/Fm). Treatments were carried out by drenching and spraying four-month-old elm greenhouse seedlings with different BABA concentrations. Plants were kept under normal watering (NW) or drought stress (DS) conditions during 8 weeks. Fv/Fm was measured at the youngest fully developed leaves after stress tests with a PAM fluorometer. Box plots present median, upper and lower quartiles; whiskers show minimum and maximum values. *n* = 5–12, ANOVA/Tukey. Statistically significant differences (*p* < 0.05) are shown with different letters above groups. *P*-values respect to controls are shown: *p < 0.05, **p < 0.01, ***p < 0.001

Leaf chlorophyll concentrations increased after treatments with BABA under both watering conditions (Fig. 2). Significant differences compared to control groups (DS, NW) were confirmed for BABA concentrations higher than 1 mM. The highest chlorophyll levels were induced by 10 mM BABA (Fig. 2). Up to 118.1% and 50.2% increased chlorophyll concentration levels were detected under NW and DS, respectively (Fig. 2). BABA showed no significant influence on the total levels of flavonoids (Supporting Information Fig. S6). However, the concentration of anthocyanins decreased after treatments at higher BABA concentrations under NW and at concentrations higher than 0.5 mM under DS (Fig. 2). Data analysis confirmed significant lower anthocyanins concentration under NW for all BABA concentrations, and for higher concentrations (≥ 5 mM) under DS (Fig. 2). The nitrogen balance index (NBI) was significantly increased after treatments at all BABA concentrations and under both watering conditions (Fig. 2). All tested BABA concentrations resulted in a significantly increased maximum quantum yield of photosystem II (Fv/Fm) between 2.50 and 5% under NW, but no significant changes were detected under DS (Fig. 3).

### BABA influenced the levels of non-canonical deoxynucleosides and ribonucleosides

**Fig. 4:**
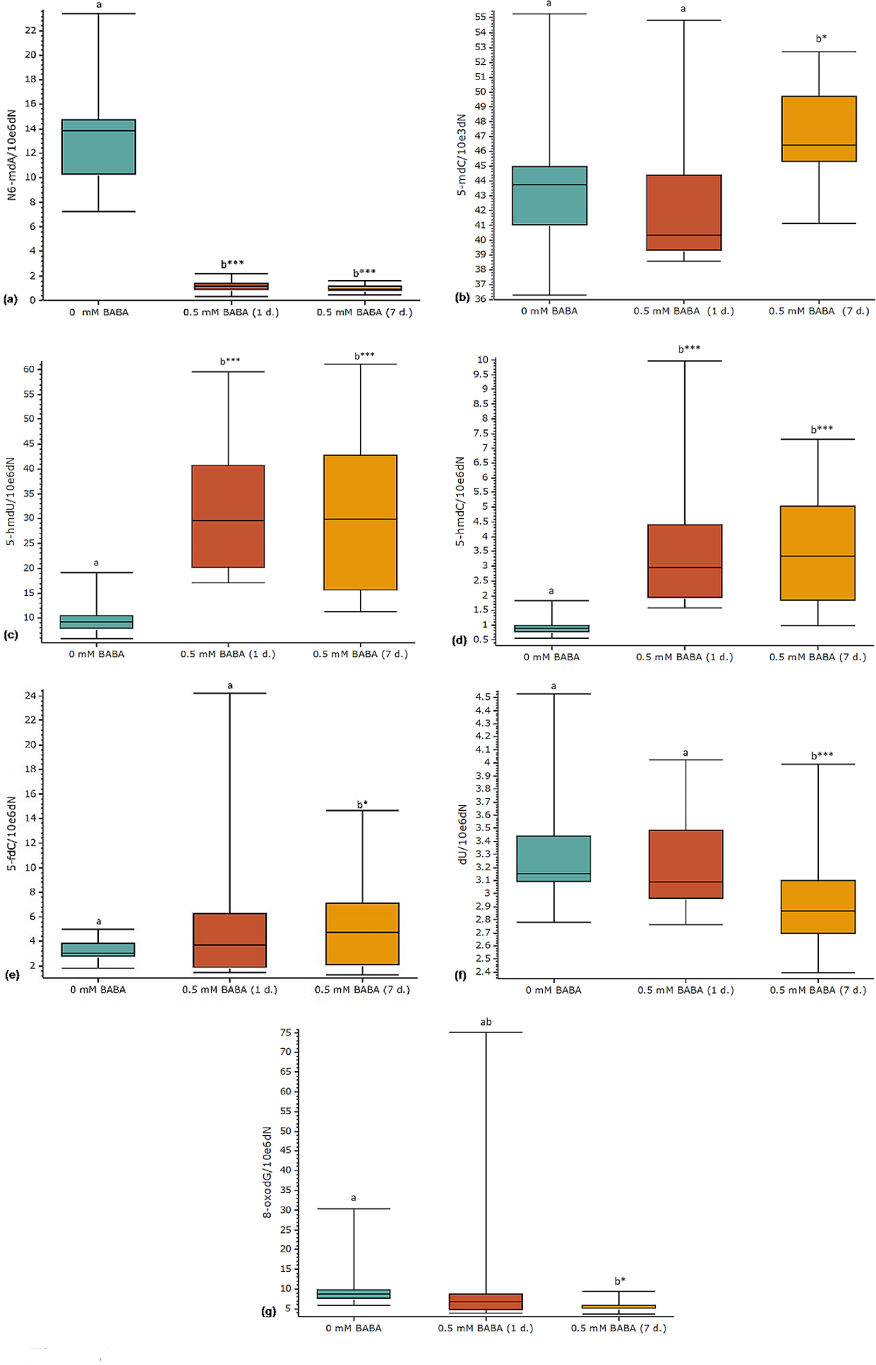
Epigenomic changes promoted by BABA. Treatments were carried out by drenching and spraying two-month-old elm greenhouse seedlings with 0.5 mM BABA. Plants were watered regularly and no drought stress was applied. The concentrations of several non-canonical deoxynucleosides, N6-mdA (**a**), 5-mdC (**b**), 5-hmdU (**c**), 5-hmdC (**d**), 5-fdC (**e**), dU (**f**), and 8-oxodG (**g**), were measured in DNA isolated from the youngest fully developed leaves in control (0 mM BABA) and treated elm seedlings (BABA 0.5 mM, 1 or 7 days after treatment). Box plots present median, upper and lower quartiles; whiskers show minimum and maximum values, *n* = 24. Statistical tests: a parametric (one-way ANOVA and multiple comparisons of Tukey) or a non-parametric (Kruskal-Wallis test and multiple comparisons of Nemenyi) test were used. Statistically significant differences (*p* < 0.05) are shown with different letters above groups. *P*-values respect to control are shown: *p < 0.05, **p < 0.01, ***p < 0.001

**Fig. 5:**
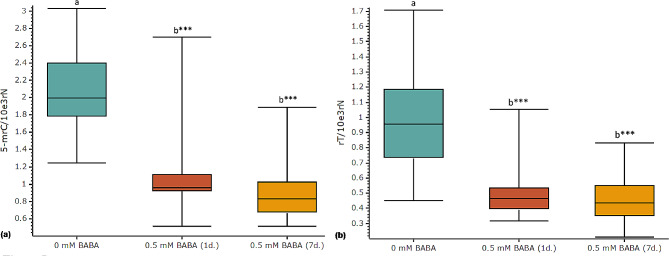
Epitranscriptomic changes promoted by BABA. Treatments were carried out by drenching and spraying two-month-old elm greenhouse seedlings with 0.5 mM BABA. Plants were watered regularly and no drought stress was applied. The concentration of two non-canonical ribonucleosides, 5-mrC (**a**) and rT (**b**), were measured in RNA isolated from the youngest fully developed leaves in control (0 mM BABA) and treated elm seedlings (BABA 0.5 mM, 1 or 7 days after treatment). Box plots present median, upper and lower quartiles; whiskers show minimum and maximum values. *n* = 10–20. Statistical tests: a parametric (one-way ANOVA and multiple comparisons of Tukey) or a non-parametric (Kruskal-Wallis test and multiple comparisons of Nemenyi) test were used. Statistically significant differences (*p* < 0.05) are shown with different letters above groups. *P*-values respect to controls are shown: *p < 0.05, **p < 0.01, ***p < 0.001

A correlation between BABA treatments and *de novo* changes at the levels of multiple non-canonical nucleosides in both DNA and RNA was found (Figs. 4 and 5). Seven different deoxynucleosides, 5-mdC, 5-hmdC, 5-fdC, 5-hmdU, 2’-deoxyuridine (dU), 8-oxo-7,8-dihydro-2’-deoxyguanosine (8-oxodG) and N6-mdA were detected in elms (Fig. 4, Supporting Information Fig. S1). The levels of 5-mdC (Fig. 4b) were 1,000-fold higher than those of all other studied deoxynucleosides (Fig. 4). Furthermore, BABA promoted concentration changes for all studied deoxynucleosides (Fig. 4). 5-MdC levels did not significantly change (3.3% decrease, *p* > 0.05) 1 day after treatments but increased 7.51% (*p* < 0.05) after 7 days (Fig. 4b). Levels of N6-mdA showed the most pronounced concentration change with a 14-fold decrease (*p* < 0.001) 1 and 7 days after treatments (Fig. 4a). Concentrations of 5-hmdC (Fig. 4d) and 5-hmdU (Fig. 4c) increased two-fold 1 and 7 days after treatments (*p* < 0.001), and 5-fdC (Fig. 4e) increased 61.66% 7 days after treatments (*p* < 0.05). The levels of 8-oxodG (Fig. 4g) and dU (Fig. 4f) decreased 41.36% (*p* < 0.05) and 10.37% (*p* < 0.001), respectively, 7 days after treatments.

Six different non-canonical ribonucleosides, 5-methylcytidine (5-mrC), ribothymidine (rT), 5-hydroxymethylcytidine (5-hmrC), N6-mrA, 5-hydroxymethyluridine (5-hmrU), and 5-formyluridine (5-frU), were detected in RNA samples (Fig. 5, Supporting Information Fig. S2). The levels of 5-mrC (Fig. 5a) and rT (Fig. 5b) decreased 49% and 54% respectively after treatments (*p* < 0.001). Levels of 5-hmrC, N6-mrA, 5-hmrU, and 5-frU were not influenced by BABA (Supporting Information Fig. S3).

## Discussion

This is the first report on BABA-induced priming in field elms. The results of drought stress tests confirmed increased stress tolerance (Figs. 1 and 2) as well as morphological (Fig. 1) and physiological (Figs. 2 and 3) changes. Many studies have shown that BABA leads to an increase in DS tolerance in several plant species (rev. in: [7]). However, no similar studies had been hitherto reported in tree species. Results of this study show that BABA promoted up to 60% PHI under DS in elms (Fig. 1). Stress-optimized regulation of plant stomata after treatments with BABA might be the reason for this growth increase [70, 71]. BABA has been found to trigger abscisic acid accumulation, which acts as a root signal, and promotes faster stomata closing in response to drought [70]. Those changes promote a lower water use, and a reduction of ROS levels, thus increasing desiccation tolerance [70].

Despite the positive effect of BABA against stress factors, its broad use for plant protection has been hampered by its growth-repressing effects under normal growth conditions [72]. In fact, some BABA concentrations can reduce vegetative growth [73, 74], as observed in field elms (Fig. 1, Supporting Information Fig. S4 and S5). It has been suggested that reduced growth might be caused in this case by disturbances of the amino acid metabolism or aspartyl tRNA synthetase (AspRS) IBI1 enzyme activity [75].

In plants, GABA is a metabolite involved in primary carbon/nitrogen (C/N) metabolism [76–79]. The NBI, a ratio between the chlorophyll and flavonoid concentration, is an indicator of C/N allocation changes caused by N-content in the soil or other factors [80–82]. The chlorophyll content is correlated with the leaf nitrogen content [83]. NBI has been considered a more robust proxy of the plant N content than chlorophyll or flavonoids alone [82, 84, 85]. NBI results showed that BABA had a clear influence on the N-content in elm leaves (Fig. 2), as only reported for GABA before [86]. This isomer of BABA has been found to connect the two major metabolic pathways of carbon and nitrogen in plants [86]. Results obtained with elms here suggest that BABA might be playing a similar role in elms and possibly in other plant species.

BABA promoted up to 118% increased chlorophyll levels in elm leaves (Fig. 2) under both DS and NW conditions. Similar results have been reported in other plant species after treatments with BABA [87] and GABA [88]. Foliar chlorophyll content under DS can increase or decrease depending on the plant species [89]. Reduction of chlorophyll concentration in some plants has been suggested to be an adaptation during DS aiming at lowering light absorption [90, 91].

BABA promoted increased Fv/Fm levels at all tested concentrations under NW but not under DS (Fig. 3). Similar Fv/Fm increases had been reported after treatments with GABA [92, 93]. In general, Fv/Fm values showed limited fluctuations under both NW and DS (Fig. 3), suggesting that field elm chloroplasts might be protected by mechanisms that dissipate excess excitation energy to prevent damage. High chlororespiration rates could be a reason behind this result in elms, as strong connections between photosynthesis and chlororespiration have been reported [94, 95].

Anthocyanin concentrations were significantly reduced by DS and BABA (Fig. 2), but the total flavonoid content did not change significantly (Supporting Information Fig. S6). Flavonoids and anthocyanins, which are a special kind of flavonoids, have been usually related to increased protection against free radicals and adjustment to oxidative stress [96, 97]. However, both increased as well as lower levels of flavonoids have been reported for different tree species under DS [98]. Similar contrasting results have been reported after treatments with BABA in plants [99–101]. These contradictory results might be due to different measuring times or species-specific factors.

In a follow-up study, treatments with 0.5 mM BABA, which strongly promoted increased PHI under DS, were chosen to investigate potential connections between BABA treatments and epigenetic changes. The presence of seven non-canonical deoxynucleosides and six non-canonical ribonucleosides was confirmed in elms. Furthermore, the broad influence of BABA on their concentrations was confirmed for the first time in a plant species (Figs. 4 and 5). Results confirmed that this BABA concentration promoted epigenomic (Fig. 4) and epitranscriptomic (Fig. 5) changes. A few studies have reported epigenetic changes after priming treatments in plant species [42, 102–104]. However, hitherto studies have been focused on 5-mdC, which is the most abundant and best-known epigenetic marker. BABA promoted increased levels of 5-mdC in elms (Fig. 4b), and potato plants [42], whereas lower levels were reported in tomato plants [103]. Those at first sight contradictory results can be explained by the nature of epigenetic changes, which are influenced by many species-specific genetic factors [55, 105].

The most pronounced changes in this study were detected at the N6-mdA levels with a 14-fold concentration decrease after treatments (Fig. 4a). N6-mdA is the most frequent epigenetic marker in bacteria [106], but it has been also reported in plants [107], tree species [108, 109], as well as in other eukaryotes [110]. N6-mdA has been related to gene expression, plant development and stress adaptation [111, 112]. Due to its low concentration in eukaryotes, and uniform genomic distribution, N6-mdA’s classification as an epigenetic marker has been questioned [113]. A toxic effect on some components of the microbiome could also explain the strong concentration decrease obtained in our study for N6-mdA, as an antimicrobial effect has been reported for BABA before [114]. The presence of N6-mdA in the nuclear DNA of animals and plants was considered a possible side product of RNA adenine methyltransferases activity [113]. According to this hypothesis changes in both N6-mdA and N6-mrA should show some correlation. However, our results indicate the opposite in elms, as stable levels of the N6-mrA (Supporting Information Fig. S3a) contrasted with strong concentration changes in N6-mdA (Fig. 4a).

The epigenetic roles of most non-canonical nucleosides in forest trees and other plant species are poorly understood [115]. Hence, most research on this topic involves other organisms. Levels of 5-hmdC increased strongly in elms after treatments (Fig. 4d). Correlation analysis of 5-hmdC and gene expression data showed an association between this deoxynucleoside and silent transposable elements in rice [116]. This is remarkable considering that the interaction between epigenetic marks and transposable elements is considered a potential key factor connecting environmental signals to DNA mutations [117].

The correlation between treatments and the increased levels of 5-hmdU in elms (Fig. 4c) might suggest a role in resistance priming, confirming reports classifying this deoxynucleoside as an epigenetic marker involved in regulatory responses to environmental changes [118]. On the other hand, 5-hmdU has also been considered either a regulatory or erroneous compound derived from oxidation reactions [119, 120]. 5-FdC levels were significantly increased seven days after treatments (Fig. 4e). 5-FdC is broadly distributed in nuclear DNA [121]. It was considered an intermediate of DNA demethylation, but results from some studies including this one suggests that it could also be an epigenetic marker involved in gene regulation [121–123].

8-OxodG and other oxidized deoxynucleosides have been associated with oxidative DNA damage and also gene regulation [124]. High levels of oxidation might activate the DNA repair systems, whereas lower oxidation levels may serve to activate other regulons [124]. Therefore, the lower levels of 8-oxodG might indicate that BABA treatments promoted a priming effect that reduced basal level of oxidative damage to DNA in elms (Fig. 4g). Furthermore, 5-mdC has also been shown to be involved in oxidative reactions in plants [120]. However, our results showed that BABA reduced oxidation and promoted higher levels of 5-mdC, 5-hmdC and 5-fdC, suggesting induction of epigenetic reprogramming in enzymatic processes rather than *via* non-specific oxidation. Uracil can be formed in DNA by spontaneous deamination of cytosine and/or arise by misincorporation of dUMP during DNA replication [125]. Regardless of the source, uracil in DNA (dU) can influence genomic stability and gene expression by interfering with promoter binding and transcription inhibition or upregulation of apoptotic proteins [126]. BABA seems to promote genome stability in elms as dU levels decreased 40% after treatments (Fig. 1f). Because both 8-oxodG and dU may be effectively removed from DNA by specific base excision repair enzymes [127], it may be hypothesized that the induced decrease may be an effect of the stimulation of the DNA repair processes.

The mechanisms involved in the alteration of the levels of most non-canonical nucleosides have not been studied yet in plants. However, first studies have reported the effect of BABA on levels of 5-mdC. BABA induced changes in DNA methylation patterns at key stress-responsive loci in potato plants [42]. Furthermore, these BABA-triggered alterations in the DNA methylome may have contributed to intergenerational priming and stress memory [42]. In this case, the expression of DNA demethylase genes like *ROS1* and *StDML2* was upregulated in a time-dependent manner upon BABA treatment, suggesting they drive the active removal of 5-mdC marks [42].

N6-mrA and N1-methyladenosine are the most abundant non-canonical ribonucleosides in plant transcriptomes [60, 128]. mRNA is less densely modified than tRNAs and rRNAs [56]. No significant changes were detected for N6-mrA, but lower concentrations were found for 5-mrC (48%) and rT (54%) in field elm RNA after treatments (Fig. 5). Several functions have been suggested for 5-mrC, including acting as a stabilizing mark important for mRNA transport throughout the plant body [129], promoting translation of heat-induced mRNAs [130], abiotic stress response [56], and promoting root development [131]. 5-MrC in mRNA is considered a new epitranscriptomic marker involved in gene regulation in plants [132, 133]. RNA 5-mrC and histone 3 lysine27 trimethylation (H3K27me3) modification are key regulators of vegetative development in *Arabidopsis* [133]. Ribothymidine may be formed in RNA by spontaneous deamination of 5-mrC, or by enzymatic methylation of rU. In many species rT is a canonical constituent of tRNA [134], responsible for stabilizing the three-dimensional structure of tRNA [135]. Anandagopu et al. [136] showed that rT sequences are not randomly distributed in protein-coding mRNA, and suggested that it is essential for production of stable and functional proteins.

The presence of microbiome DNA and RNA poses a challenge for all methods of epigenetic research, including the mass spectrometry approach used in this study. Microbiome DNA has been also reported in reference genomes, and the *in silico* elimination of it still has many drawbacks [137–139]. On the other hand, plants have complex relations with their endogenous and exogenous microbiomes which are poorly understood. Furthermore, epigenetic changes in the microbiome can have implications for plant development and stress resistance [140].

All eukaryotes are actually complex “holobiont” communities with all their genes building the “hologenome” [141], “holoepigenome” and “holoepitranscriptome”. The production of microbial low-molecular-weight molecules, such as methyl or acetyl groups, phosphatases, methyltransferases, biotin, and eukaryote-like serine/threonine kinases, may directly affect epigenetic processes in eukaryotes [142]. The strong decrease in N6-mdA levels detected after treatments (Fig. 4a) could be related to the field elm genome, its microbiome or even both. Epigenetic changes in any part of the microbiome can induce multiple changes in plants and vice versa [140]. Therefore, approaches that keep plant and microbiome DNA or RNA together represent valuable tools for epigenetic research as their results better reflect the state of plants in nature.

The fields of applied epigenetics and microbiomics are evolving rapidly. It is now clear that epigenetic mechanisms have also provided useful variability during crop varietal selection [104, 143–146]. Furthermore, studies have shown that crop domestication and breeding have inadvertently changed the microbial communities of crops [147, 148], and that the microbiome composition is a trait that can be bred [149]. Therefore, epigenetics and microbiomics have great potential for resistance induction in forest tree species, as crossing breeding is hampered in this case by their prolonged non-reproductive phase.

The application of priming approaches in long-living plant species requires long-term improved stress tolerance. This is a critical aspect for this and most similar studies as it has been found that the priming effect is the norm in the short-term, but its persistence over multiple growing seasons or generations is less common [7]. Epigenetic modifications are thought to be key mechanisms underlying transgenerational priming in plants [45–54]. These epigenetic marks can be mitotically and meiotically heritable, allowing for the inheritance of primed states to offspring. However, the stability and heritability of priming-induced epigenetic changes remains an open question. Reversion of epigenetic marks and dilution over successive generations may limit the durability of priming in long-lived plants. Strategies to stabilize priming-associated epigenetic changes, such as targeting key transcriptional regulators, may be necessary to achieve persistent priming.

Plants, like all other living organisms, have developed some form of memory that enables them to adapt to changing environmental conditions [50, 150–152]. Epigenetic factors and phytohormones [50] have been shown to be involved in plant memory [45–47]. However, other mechanisms, similar to the neuronal process described for animals, have been also proposed [48, 49]. In addition to some plant-specific transmitters, plants use other transmitters, receptors and interacting molecules in their rapid cell-to-cell communication that are also present in neuronal tissues, e.g. glutamate, ionotropic glutamate receptors, glycine and aminobutyric acid [48]. Both GABA [13, 48, 153] and BABA [12, 154] are rapidly produced under stress conditions and are transported from cell to cell across plant tissues. Learning in animals is based on the endocytic recycling of GABA and glutamate receptor *via* synaptic plasticity [155]. Glutamine inhibits BABA-induced priming and stress resistance in plants [74]. More research is still required to define the specific roles of both aminobutyric acid isomers on stress resistance and stress memory, and the role of the microbiome as potential GABA and BABA provider in plants, as is the case for GABA in animals [156].

Prolonged non-reproductive phases lasting even decades are still the main obstacle for resistance induction in forest tree species. Therefore, alternative methods are urgently required to deal with the growing number of plagues and illnesses threatening forests worldwide. The characterization of the molecular systems involved in plant stress memory is a step forward in the development of new reliable approaches for resistance induction in trees.

## Conclusions

The presented results confirmed the influence of BABA on development, physiology, and stress tolerance in field elms. However, the most important results are related to the broad epigenetic changes promoted by this amino acid, which involve both DNA and RNA. Our findings confirm that not only the well-known epigenetic markers in plants, such as 5-mdC and 5-mrC, but also several other non-canonical nucleosides are influenced by BABA. The significant effect on the levels of N6-mdA, the main bacterial epigenetic marker, is particularly noteworthy. In this case, the question arises as to whether this effect is due to epigenetic changes in the microbiome or plant genome, or both. The plant phenotype is the result of complex interactions between the plant’s DNA, the microbiome, and the environment. We propose that different types of epigenetic changes in the plant and microbiome may play important roles in the largely unknown memory process that enables plants to adapt faster to changing environmental conditions.

## Materials and methods

### Plant material and priming treatments

Field elm (*Ulmus minor* Mill.) seeds, provenience 4 (SP/27/B130), were obtained from the Forest Gene Resources Center (FOGZ, Lippstadt/Germany). Studies were carried out at the Institute of Forest Genetics in Grosshansdorf, Germany (Latitude: 53° 39′ 42.5952″ N, Longitude: 10° 15′ 12.7764″ E).

Elm plants were treated with BABA (Sigma/Germany, Art. Nr. A44207-25G). Germinated seeds were transplanted into pots containing fertilized soil. After several repotting steps plants were grown in 3 L pots for the planned DS tests. Plants were grown in growth chambers (Weiss Technik, Reiskirchen, Germany) under the following culture conditions: light period 18/6 h (day/night), light irradiance 300 μmol m^− 2^ s^− 1^ (lamps Phillips TLM 140 W/33RS, Amsterdam, The Netherlands), relative humidity 60% and temperature 22/19°C. After a culture period of two months in growth chambers, plants were transferred to a standard greenhouse under natural conditions.

### Drought stress tests

Four-month-old untreated plants were randomly distributed between groups for treatment with one of five different BABA concentrations (0/0.5/1/5/10 mM). The BABA solutions were prepared in tap water. BABA was applied to plants, using both soil drenching and spraying, two days before starting DS tests. Plants were kept in a standard greenhouse under natural conditions.

Eight-week long DS tests were carried out in a greenhouse. Plants were watered using a drip system designed for this aim. Water requirements were stablished after weighting a selected group of the treated plants. The plants were distributed in a greenhouse under natural temperature and light, using a randomized block design. Two blocks were built based on the tested watering conditions, i.e., 70% and 20% water capacity (WC). In each block randomized plant positions were re-randomized regularly. The WC was calculated using saturated soil weight and dry weight at three exemplary pots [157]. Several parameters were measured before and at the end of the DS tests. Leaf parameters were measured at the uppermost fully expanded leaves of plants. Dependent variables were plant height increase (PHI = final height - initial height), plant height (PH: final height), content of leaf pigments (chlorophyll, flavonoids, anthocyanin), nitrogen balance index (NBI), and maximum quantum yield of photosystem II (Fv/Fm). Independent variables were BABA concentrations (0/0.5/1/5/10 mM), and watering conditions (70% or 20% of the maximal soil WC). Leaf pigment concentration and NBI were determined using a non-destructive Dualex device [158]. Fv/Fm was measured after 30 min of dark adaptation [159] using a Pulse Amplitude Modulation fluorometer (PAM 2100, Walz, Effeltrich, Germany). Maximum fluorescence level (Fm) was measured with 0.8 s lasting of saturating pulses at 4,000 μmol m^− 2^ s^− 1^.

### Epigenetic changes promoted by BABA

Priming treatments were carried out by drenching and spraying two-month-old elm greenhouse seedlings with 0.5 mM BABA. Plants were watered regularly and no drought stress was applied. This highly effective BABA concentration was selected based on previous phenotyping results. Control plants were similarly treated with the same tap water used for BABA solutions. Plants from the two treatment groups were treated 1 and 7 days before the sampling day. Samples of treated and control plants (*n* = 10–24) were taken on the same day from the uppermost fully developed leaves. Samples were frozen using liquid nitrogen and stored at -70 °C until DNA and RNA isolation.

### DNA and RNA isolation and hydrolysis to nucleosides

DNA was isolated from leaves with the GeneMATRIX Plant & Fungi DNA Purification Kit (EURx) (Cat. Nr. 3595) and the Lyse CT buffer (Cat. Nr. 0324) following the manufacturer’s instructions. RNA isolation was carried out with the GeneMATRIX Universal RNA Purification Kit (EURx) (Cat. Nr. 3598) and Lyse Buffer PVP (Cat. Nr. E0291) according to the manufacturer’s instructions. Nucleic acids (NA) hydrolysis was performed using a modified method described by Starczak et al. [160]. Briefly, NA samples were completely dried in a SpeedVac system. Next, the pellet was dissolved in 50 μL of MilliQ-grade deionized water and mixed with 50 μL of NP1 buffer (200 mM ammonium acetate (Sigma-Aldrich, Cat. Nr 73594), 0.2 mM ZnCl_2_ (POCH, Cat. Nr 264170113); pH 4.6). Nuclease P1 (100 U, New England Biolabs, Cat. Nr M0660S) and tetrahydrouridine (10 mg/ml, Merck Cat. Nr 584222) were added to the mixture and incubated at 37 °C overnight. Subsequently, 13 μL of 10% (v/v) NH_4_OH (J.T.Baker Cat. Nr 4807-05) and 12 U of Shrimp Alkaline Phosphatase (rSAP, New England Biolabs, Cat. Nr M0371L) were added to each sample following 2 h incubation at 37 °C. All hydrolysates were ultrafiltered prior to injection and concentrated in a SpeedVac to a final volume of 10 μL. All modifications were quantified in the form of the corresponding ribo- or deoxynucleosides using mass spectrometry.

### Mass spectrometric quantification of non-canonical deoxynucleosides in DNA

DNA hydrolysates were spiked with a solution of internal standard in volumetric ratio 4:1, to a concentration of 50 fmol/μL of [^13^C_10_, ^15^N_2_]-5-mdC, [D_3_]-5-hmdC, [^13^C_10_, ^15^N_2_]-5-fdC, [^13^C_10_, ^15^N_2_]-5-hmdU, [^13^C, ^15^N_2_]-dU, [^15^N_5_]-8-oxodG and 5 fmol/μL of [D_3_]-N6-mdA, and analyzed using isotope-dilution automated online two-dimensional ultra-performance liquid chromatography with tandem mass spectrometry (2D-UPLC-MS/MS).

Chromatographic separation was achieved using a method described by Starczak et al. [160] based on a 2D-UPLC system with photo-diode array detector for the first dimension chromatography (used for the quantification of canonical deoxynucleosides) and tandem quadrupole mass spectrometer (Xevo TQ-XS, Waters) using the following columns: Waters Cortecs T3 column (150 mm×3 mm, 1.6 μm) with a precolumn at the first dimension, a Waters X-select C18 CSH (100 mm×2.1 mm, 1.7 μm) at the second dimension and Waters X-select C18 CSH (20 mm×3 mm, 3.5 μm) as a trap/transfer column. At the first dimension a flow rate of 0.5 mL/min was used, with an injection volume of 2 μL and gradient elution for 10 min using a mobile phase of 0.05% acetate (A) and acetonitrile (B) (0.7-5% B for 5 min, followed by the column washing with 30% acetonitrile and re-equilibration with 99% A for 3.6 min). At the second dimension the flow rate was 0.35 mL/min in a gradient elution for 10 min using a mobile phase of 0.01% acetate (A) and methanol (B) (1–50% B for 4 min, isocratic flow of 50% B for 1.5 min, and re-equilibration with 99% A up to the next injection).

All samples were analyzed in three to five technical replicates and the technical mean was used for further calculation. Transition patterns for all the analyzed compounds along with specific detector settings are shown in Supplementary Table S1. The quantities of canonical deoxynucleosides were determined by UV detection at 260 nm for 2’-deoxythymidine (dT), and at 280 nm for 2’-deoxyguanosine (dG) and 5-mdC. Total deoxynucleosides amount (dN) calculated as doubled sum of dT and dG was used as a reference for quantitative expression of modified ones.

### Mass spectrometric quantification of non-canonical ribonucleosides in RNA

RNA hydrolysates were spiked with a solution of internal standard in volumetric ratio 4:1, to a concentration of 100 fmol/μL of [^13^C, D_3_]− 5-mrC, [^13^C, D_2_]-5-hmrC, [D_3_]-N6-mrA, [^13^C_5_]-rT, [^13^C_5_]-5-hmrU, and [^13^C_5_]-5frU.

Chromatographic separation was performed using a UPLC system with photo-diode array detector (used for the quantification of canonical ribonucleosides) and tandem quadrupole mass spectrometer (Xevo TQ-XS, Waters) using Waters Cortecs T3 column (150 mm×3 mm, 1.6 μm) with a precolumn. Flow rate was 0.5 mL/min, with an injection volume 2 μL and gradient elution was applied for 10 min using a mobile phase 0.05% acetate with 5μM ammonium formate (A) and acetonitrile (B) (0.7% B for 0.5 min, followed by gradient (0.7–50% B) for 4.5 min, the column was washed with 50% acetonitrile for 2.2 min and re-equilibrated with 99% A for 2.8 min. All samples were analyzed in three to five technical replicates and the technical mean was used for further calculation. Transition patterns for all the analyzed compounds along with specific detector settings are shown in the Supplementary Table S2.

The quantities of canonical ribonucleosides were determined by UV detection at 260 nm for uridine (rU), 280 nm for guanosine (rG), adenosine (rA) and cytidine (rC). Total ribonucleosides amount (rN), calculated as the sum rU + rG + rA + rC, was used as a reference for quantitative expression of non-canonical ribonucleosides in RNA.

### Statistical data analysis and artwork

Data were analyzed with MaxStat 3.60 and STATeasy software. Data distribution (Kolmogorov-Smirnov test) and variance (Bartlett test) were evaluated for selection of either a parametric (one-way ANOVA and multiple comparisons of Tukey) or a non-parametric (Kruskal-Wallis test and multiple comparisons of Nemenyi) approach. Significant differences (*p* < 0.05) are shown in figures with different letters; *p* values (*p < 0.05, **p < 0.01 and ***p < 0.001) indicate significant differences respect to the control group. All experiments were repeated at least twice, and treatment groups consisted of up to 30 biological replicates. Figures were prepared using MaxStat 3.60, MS PowerPoint, MS Paint and GIMP 2.10.14.

### Electronic supplementary material

Below is the link to the electronic supplementary material.

Supplementary Material 1

Supplementary Material 2

## Data Availability

The file “Original Data” is available at BMC Plant Biology online.

## Abbreviations

2D-UPLC-MS/MS: Two-dimensional ultra-performance liquid chromatography with tandem mass spectrometry
5-fdC: 5-Formyl-2’-deoxycytidine
5-frU: 5-Formyluridine
rU: Uridine
5-hmdC: 5-(Hydroxymethyl)-2’-deoxycytidine
5-hmdU: 5-(Hydroxymethyl)-2’deoxyuridine
5-hmrC: 5-Hydroxymethylcytidine
5-hmrU: 5-Hydroxymethyluridine
5-mdC: 5-Methyl-2’-deoxycytidine
5-mrC: 5-Methylcytidine
8-oxodG: 8-Oxo-7,8-dihydro-2’-deoxyguanosine
AABA: α-Aminobutyric acid
BABA: β-Aminobutyric acid
dG: 2’-Deoxyguanosine
dN: Total deoxynucleosides amount
DS: Drought stress
dT: 2’-Deoxythymidine
dU: 2’-Deoxyuridine
Fv/Fm: Maximum quantum yield of photosystem II
GABA: γ-aminobutyric acid
MS: Mass spectrometry
N6-mdA: N6-methyl-2’-deoxyadenosine
N6-mrA: N6-methyladenosine
NBI: Nitrogen balance index
NW: Normal watering
PH: Plant height
PHI: Plant height increase
rA: Adenosine
rC: Cytidine
rG: Guanosine
rN: Total ribonucleosides amount
rT: Ribothymidine
UPLC: Reversed-phase ultra-performance liquid chromatography
