# Supplementary material for: β-Aminobutyric acid promotes stress tolerance, physiological adjustments, as well as broad epigenetic changes at DNA and RNA nucleobases in field elms (Ulmus minor)
Source: BMC Plant Biol. 2024 Aug 15;24:779. doi: 10.1186/s12870-024-05425-6 (PMC11325618; doi:10.1186/s12870-024-05425-6)
Supplement: Supplementary file 1 — Supplementary Material 1 [file 12870_2024_5425_MOESM1_ESM.docx]

## *BMC Plant Biology*

## Supporting Information

Article title: β-aminobutyric acid promotes stress tolerance, physiological adjustments, as well

as broad epigenetic changes at DNA and RNA nucleobases in field elms (*Ulmus minor*)

Authors: Hans Hoenicka, Susanne Bein, Marta Starczak, Wolfgang Graf, Dieter Hanelt, Daniel Gackowski

The following Supporting Information is available for this article:

**Fig. S1. Chromatograms of deoxynucleosides in field elm DNA**

N6-mdA (a), 5-hmdC (b), 5-fdC (c), 5-hmdU (d), dU (e), 8-oxodG (f), 5-mdC (g), dG (h), dT (i).

Methods used: mass spectrometry (a, b, c, d, e, f), UV detection (g, h, i).


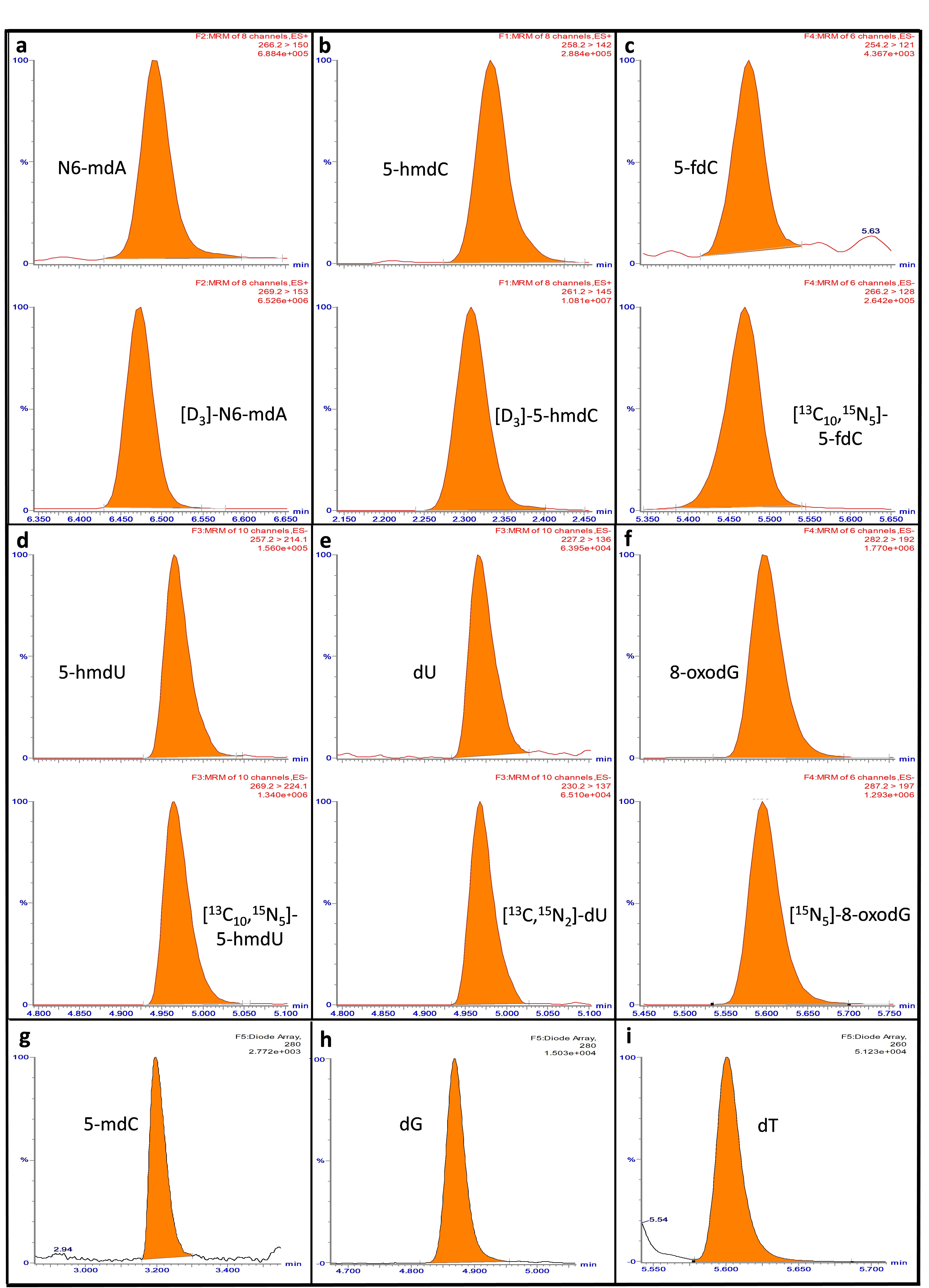

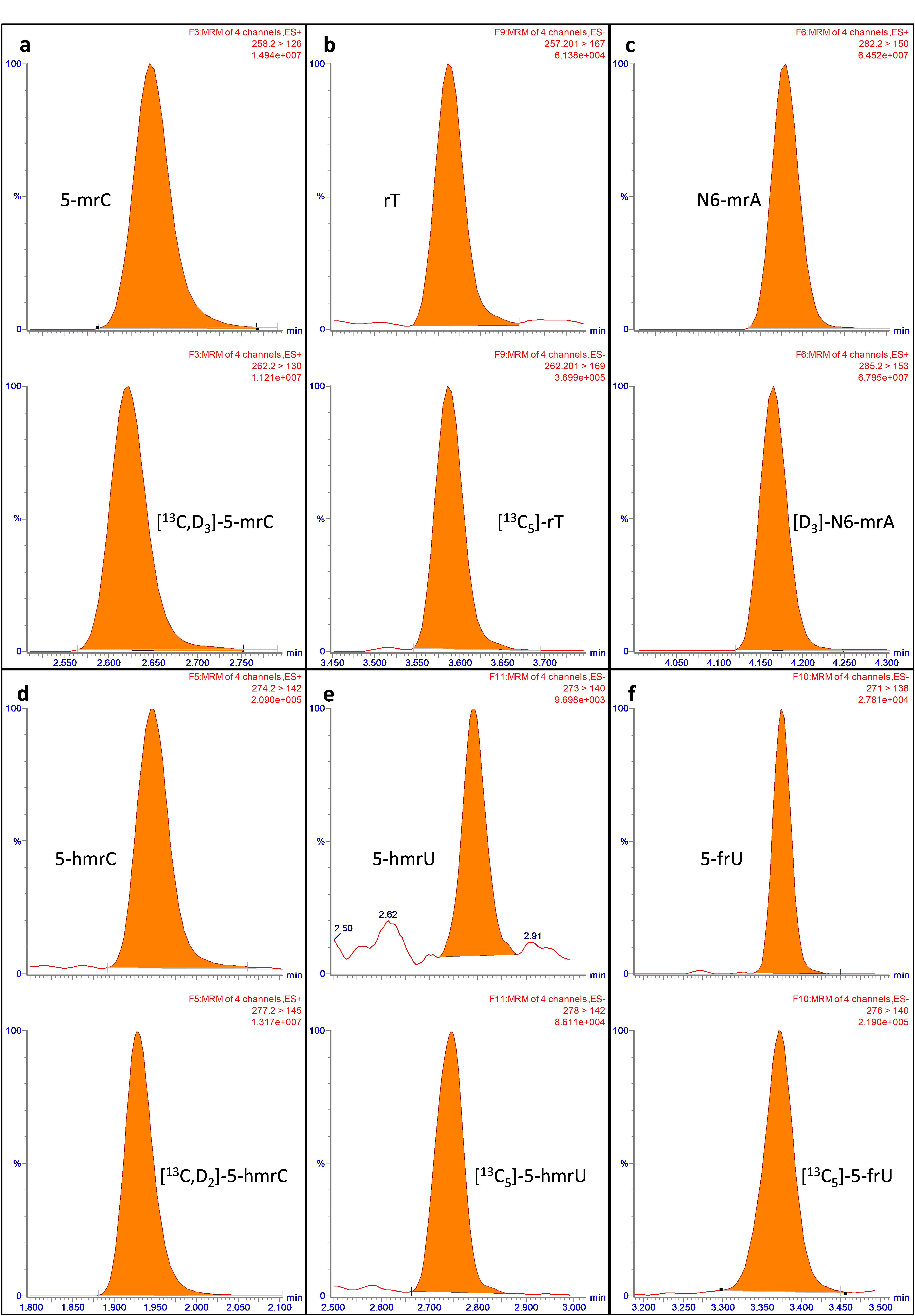


**Fig. S2. Chromatograms of ribonucleosides in field elm RNA**

5-mrC (a), rT (b), N6-mrA (c), 5-hmrC (d), 5-hmrU (e), 5-frUn (f)

Method used: mass spectrometry.


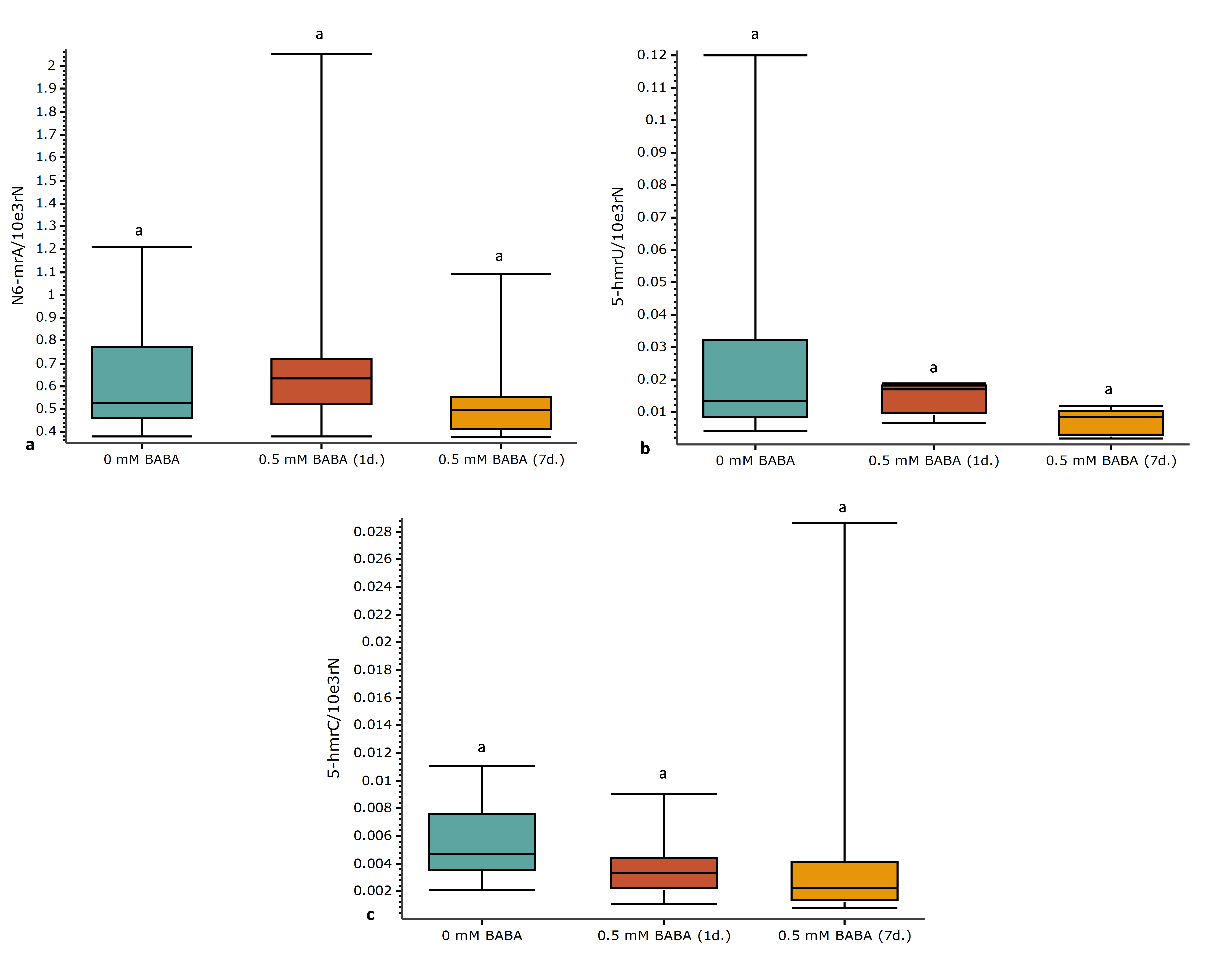


**Fig. S3. Ribonucleosides showing stable concentrations after BABA treatments.**

The concentration of non-canonical ribonucleosides, N6-mrA (a), 5-hmrU (b), and 5-hmrC (c), measured (0,1,7 days) in control and treated seedlings (BABA 0.5 mM), were not significantly influenced by BABA. Box plots present median, upper and lower quartiles; whiskers show minimum and maximum values. n=10-20, ANOVA/Tuckey or Kruskal Wallis/Nemenyi test. No statistically significant differences were found.


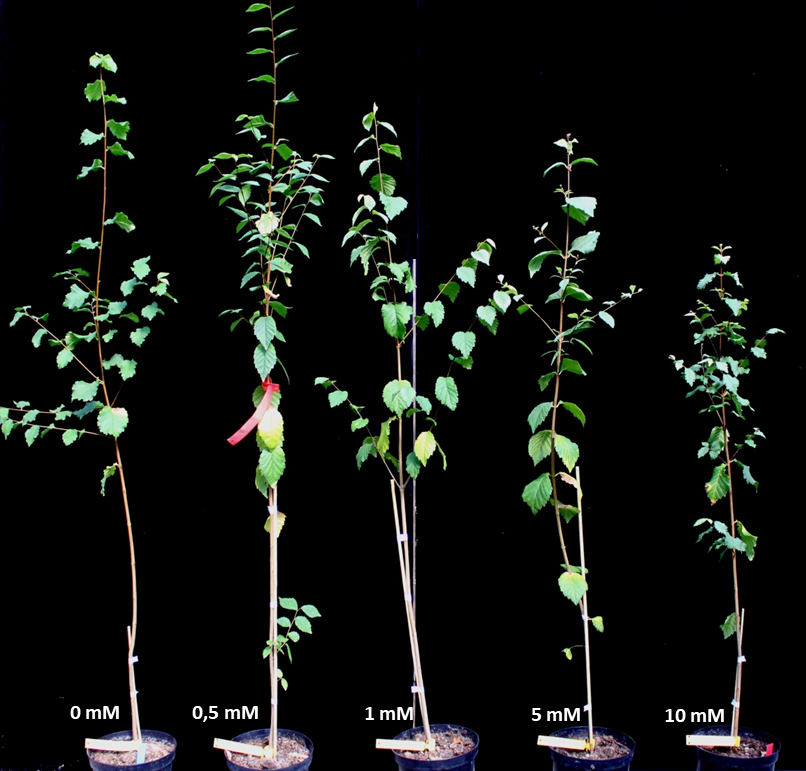


DS


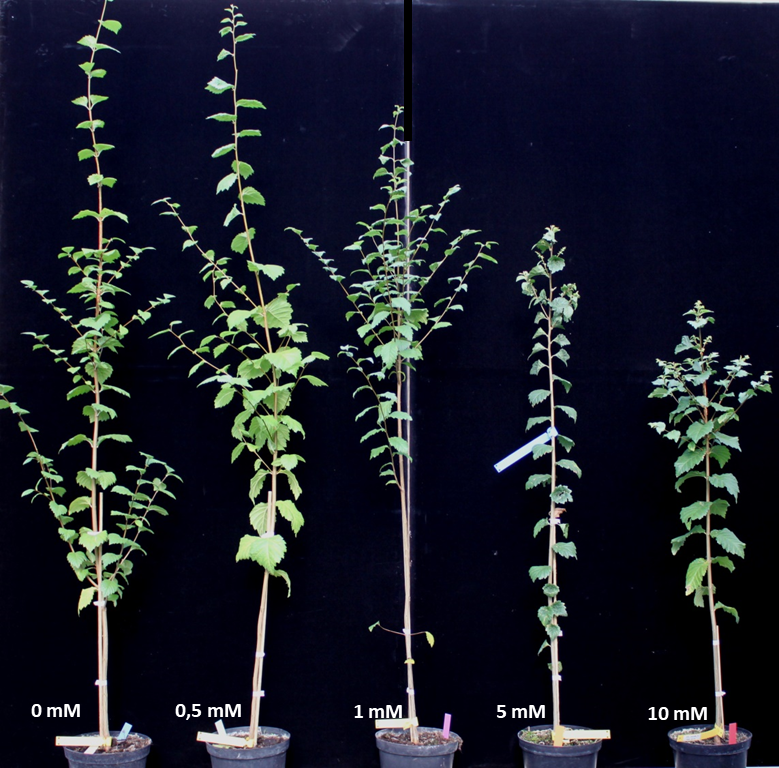


NW

**Fig. S4. Effect of BABA and watering conditions on field elm development.**

BABA concentrations: 0, 0.5, 1, 5, 10 mM. Watering conditions: 20% (DS) and 70% (NW) of soil water capacity. Plants are shown 2 months after BABA treatments and 6 months after seed germination.


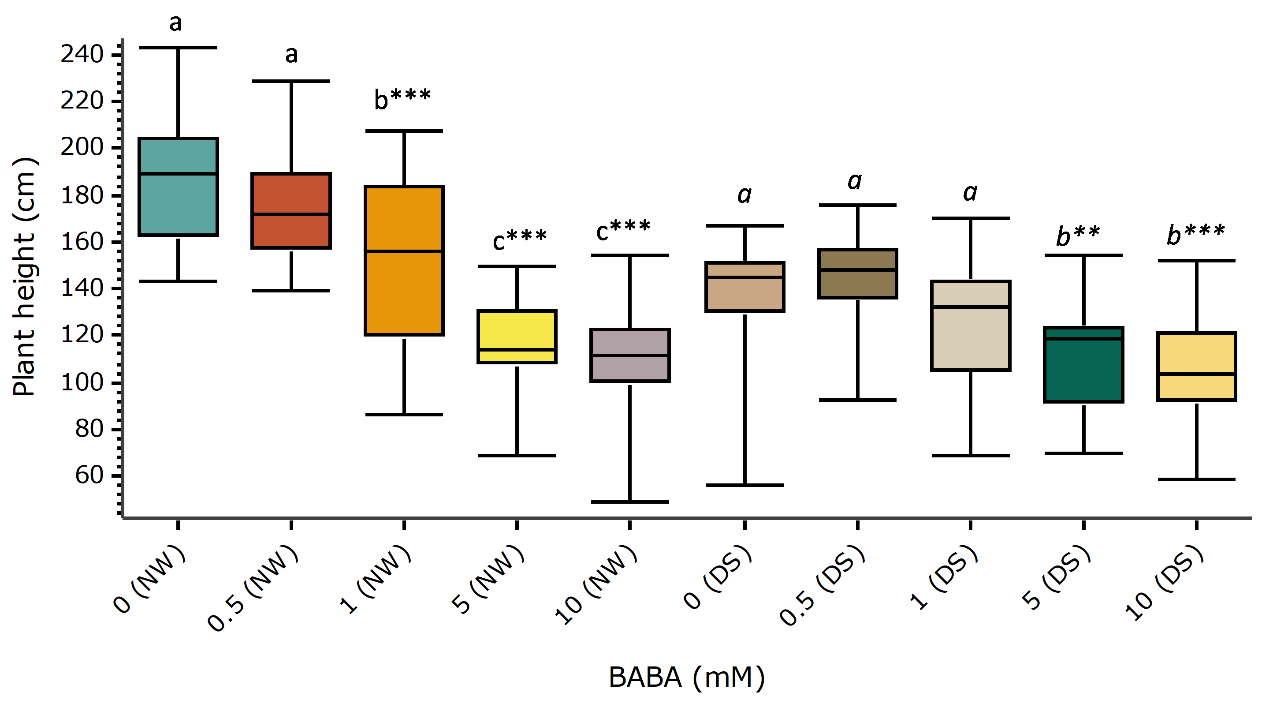


**Fig. S5. Higher BABA concentrations influenced plant height.**

Plants were measured at the end of the experiments, namely six months after germination and two months after priming with BABA and drought stress tests. Seedlings were grown under normal watering (NW) or drought stress (DS) conditions. Box plots present median, upper and lower quartiles; whiskers show minimum and maximum values, n=30. Statistical tests: one-way ANOVA and multiple comparisons of Tuckey. Comparisons were made between priming groups and the corresponding watering control, 0 (NW) or 0 (DS). Statistically significant differences (p<0.05) are shown with different letters above groups. P-values respect to controls are shown: *p<0.05, **p<0.01, ***p<0.001.


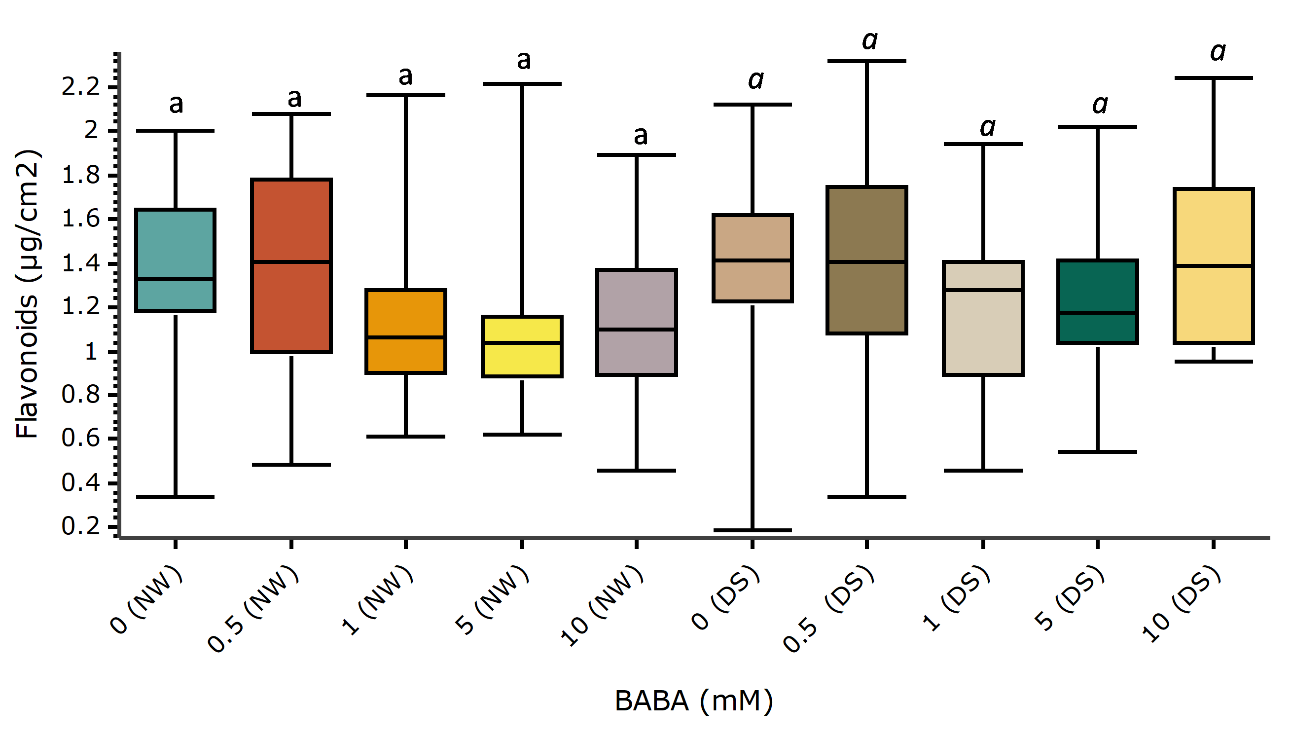


**Fig. S6. BABA did not influence total flavonoid concentration.**

Results are based on measurements of the epidermal UV absorbance with a Dualex device. Uppermost leaves were measured at the end of the experiments. Seedlings were grown under normal watering (NW) or drought stress (DS) conditions. Box plots present median, upper and lower quartiles; whiskers show minimum and maximum values, n=30. Statistical tests: one-way ANOVA and multiple comparisons of Tuckey. Comparisons were made between priming groups and the corresponding watering control group, 0 (NW) or 0 (DS). No statistically significant differences were found

| **compound name** | **ionization mode** | **nominal molecular mass (Da)** | **pseudomolecular ion formulation** | **nominal parent ion (Da)** | **nominal daughter ion (Da)** | **ESI**  **capillary (kV)** | **ESI**  **cone (V)** | **collision**  **energy (eV)** | **standard source** |
| --- | --- | --- | --- | --- | --- | --- | --- | --- | --- |
| 5-(hydroxymethyl)-2'-deoxycytidine | positive | 257 | [M+H]+ | 258 | 124 | 1.2 | 15 | 10 | Berry & Associates |
| [D_3_]-5-(hydroxymethyl)-2'-deoxycytidine | positive | 260 | [(M+3)+H]+ | 261 | 127 | 1.2 | 15 | 10 | Toronto Research Chemicals |
| 5-formyl-2'-deoxycytidine | negative | 255 | [M-H]- | 254 | 121 | 3.5 | 28 | 18 | Berry & Associates |
| [^13^C_10_, ^15^N_2_]-5-formyl-2'-deoxycytidine | negative | 267 | [(M+12)-H]- | 266 | 128 | 3.5 | 28 | 18 | own synthesis |
| 5-(hydroxymethyl)-2'-deoxyuridine | negative | 258 | [M-H]- | 257 | 124 | 3.5 | 20 | 15 | Berry & Associates |
| [^13^C_10_, ^15^N_2_]-5-(hydroxymethyl)-2'-deoxyuridine | negative | 270 | [(M+12)-H]- | 269 | 131 | 3.5 | 20 | 15 | own synthesis |
| 2'-deoxyuridine | negative | 228 | [M-H]- | 227 | 184 | 3.5 | 20 | 12 | Sigma-Aldrich |
| [^13^C, ^15^N_2_]-2'-deoxyuridine | negative | 231 | [(M+3)-H]- | 230 | 185 | 3.5 | 20 | 12 | Medical Isotopes |
| 8-oxo-7,8-dihydro-2'-deoxyguanosine | negative | 283 | [M-H]- | 282 | 192 | 1.2 | 20 | 15 | Jena Bioscience |
| [^15^N_5_]-8-oxo-7,8-dihydro-2'-deoxyguanosine | negative | 288 | [(M+5)-H]- | 287 | 197 | 1.2 | 20 | 15 | Cambridge Isotope Laboratories |
| 5-methyl-2'-deoxycytidine | positive | 241 | [M+H]+ | 242 | 126 | 1.2 | 12 | 18 | Jena Bioscience |
| [^13^C_10_, ^15^N_2_]-5-methyl-2'-deoxycytidine | positive | 253 | [(M+12)+H]+ | 254 | 133 | 1.2 | 12 | 18 | own synthesis |
| N6-methyl-2’-deoxyadenosine | positive | 265 | [M+H]+ | 266 | 150 | 3.5 | 15 | 15 | Toronto Research Chemicals |
| [D_3_]-N6-methyl-2’-deoxyadenosine | positive | 268 | [(M+3)+H]+ | 269 | 153 | 3.5 | 15 | 18 | Toronto Research Chemicals |

**Table S1.** Transition patterns, specific detector settings and sources of standards for analyzed deoxynucleosides.

| **compound name** | **ionization mode** | **nominal molecular mass (Da)** | **pseudomolecular ion formulation** | **nominal parent ion (Da)** | **nominal daughter ion (Da)** | **ESI**  **capillary (kV)** | **ESI**  **cone (V)** | **collision**  **energy (eV)** | **standard source** |
| --- | --- | --- | --- | --- | --- | --- | --- | --- | --- |
| 5-methylcytidine | positive | 257 | [M+H]+ | 258 | 126 | 3 | 18 | 14 | Toronto Research Chemicals |
| [^13^C, D_3_]-5-methyl-2'-deoxycytidine | positive | 261 | [(M+4)+H]+ | 262 | 130 | 3 | 18 | 14 | Toronto Research Chemicals |
| 5-hydroxymethylcytidine | positive | 273 | [M+H]+ | 274 | 142 | 3 | 18 | 12 | Toronto Research Chemicals |
| [^13^C, D_2_]-5-hydroxymethylcytidine | positive | 276 | [(M+3)+H]+ | 277 | 145 | 3 | 18 | 12 | Toronto Research Chemicals |
| rybothymidine | negative | 258 | [M-H]- | 257 | 167 | 3 | 25 | 13 | Sigma-Aldrich |
| [^13^C_5_]- rybothymidine | negative | 263 | [(M+5)-H]- | 262 | 169 | 3 | 25 | 13 | Medical Isotopes |
| 5-hydroxymethyluridine | negative | 274 | [M-H]- | 273 | 140 | 3 | 20 | 15 | Sigma-Aldrich |
| [^13^C_5_]-5-hydroxymethyluridine | negative | 279 | [(M+5)-H]- | 278 | 142 | 3 | 20 | 15 | own synthesis |
| 5-formyluridine | negative | 272 | [M-H]- | 271 | 138 | 3 | 20 | 15 | own synthesis |
| [^13^C_5_]-5-formyluridine | negative | 277 | [(M+5)-H]- | 276 | 140 | 3 | 20 | 15 | own synthesis |
| N6-methyladenosine | positive | 281 | [M+H]+ | 282 | 150 | 3 | 10 | 18 | Toronto Research Chemicals |
| [D_3_]-N6-methyladenosine | positive | 284 | [(M+3)+H]+ | 285 | 153 | 3 | 10 | 18 | Toronto Research Chemicals |

**Table S2.** Transition patterns, specific detector settings and sources of standards for analyzed rybonucleosides.
